# Radiomic Analysis of Quantitative T2 Mapping and Conventional MRI in Predicting Histologic Grade of Bladder Cancer

**DOI:** 10.3390/jcm12185900

**Published:** 2023-09-11

**Authors:** Lei Ye, Yayi Wang, Wanxin Xiang, Jin Yao, Jiaming Liu, Bin Song

**Affiliations:** 1 Department of Radiology, West China Hospital, Sichuan University, Chengdu 610041, China; yelei_radiology@foxmail.com (L.Y.); wang-yayi@foxmail.com (Y.W.); xiangwx1022@foxmail.com (W.X.); songlab_radiology@163.com (B.S.); 2 Department of Ophthalmology, West China Hospital, Sichuan University, Chengdu 610041, China; 3 Department of Urology, Institute of Urology, West China Hospital, Sichuan University, Chengdu 610041, China

**Keywords:** bladder cancer, histologic grade, artificial intelligence, multiparametric MRI, radiomic analysis, prediction

## Abstract

We explored the added value of a radiomic strategy based on quantitative transverse relaxation (T2) mapping and conventional magnetic resonance imaging (MRI) to evaluate the histologic grade of bladder cancer (BCa) preoperatively. Patients who were suspected of BCa underwent pelvic MRI (including T2 mapping and diffusion-weighted imaging (DWI) before any treatment. All patients with histological-proved urothelial BCa were included. We constructed different prediction models using the mean signal values and radiomic features from both T2 mapping and apparent diffusion coefficient (ADC) maps. The diagnostic performance of each model or parameter was assessed using receiver operating characteristic curves. In total, 92 patients were finally included (training cohort, *n* = 64; testing cohort, *n* = 28); among these, 71 had high-grade BCa. In the testing cohort, the T2-mapping radiomic model achieved the highest prediction performance (area under the curve (AUC), 0.87; 95% confidence interval (CI), 0.73–1.0) compared with the ADC radiomic model (AUC, 0.77; 95%CI, 0.56–0.97), and the joint radiomic model of 0.78 (95%CI, 0.61–0.96). Our results demonstrated that radiomic mapping could provide more information than direct evaluation of T2 and ADC values in differentiating histological grades of BCa. Additionally, among the radiomic models, the T2-mapping radiomic model outperformed the ADC and joint radiomic models.

## 1. Introduction

Bladder cancer (BCa) is one of the most expensive malignancies to manage owing to its high-recurrence rate [1]. BCa can be histologically classified into high- and low-grade cancers. High-grade BCa accounts for one-third of non-muscle-invasive BCa (NMIBC), which can progress to muscle-invasive BCa (MIBC) and metastasize in approximately 20–25% of patients, while low-grade BCa often has an indolent natural history [2]. Management of BCa is based on the histology, grade, and depth of muscle invasion, and these factors play a crucial role in determining the prognosis and treatment strategies for patients with BCa [3,4]. Thus, transurethral resection of the bladder tumor (TURBT) is the recommended approach for confirming the grade and depth of muscular invasion in BCa [5]. To reduce the probability of recurrence, patients with high-grade tumors require intravesical Bacillus Calmette–Guérin (BCG) instillations for 1–3 years [2]. However, TURBT is associated with significant risks of understaging and undergrading, and not removing the entire tumor; accordingly, this may require repeated biopsies [5,6]. However, a second TURBT is not recommended for patients with pathologically reported low-grade NMIBC, even when the tumor specimen is insufficient; thus, it may lead to delays or disallow patients from additional BCG therapies. Therefore, it would be of great benefit to explore a noninvasive method to predict the pathological grade and T stage preoperatively. This would help prevent patients from undergoing repeat surgeries and guarantee timely BCG treatment. Considering that multiparametric magnetic resonance imaging (mpMRI) has been extensively used for the local staging of BCa [7,8], it is worth using mpMRI for the preoperative evaluation of the pathological grade.

Recently, mpMRI has demonstrated its emerging role in differentiating malignant heterogeneity in various organs [9,10,11]. Some MRI sequences enable radiologists to per-form noninvasive quantitative assessments of lesions. For instance, transverse relaxation (T2) mapping is a quantitative technique based on voxel-wise evaluation of proton spin–spin relaxation times that allows for noninvasive visualization and quantification of tissue composition, particularly free-water content [12]. Although initially developed for the assessment of myocardial injury [13], T2-weighted parametric mapping is increasingly being applied to other organs such as the brain, liver, and kidney [12,14,15]. In the context of this study, it is extensively reported that tumors with higher cellularity demonstrate a corresponding lower signal in T2 maps as a result of a reduction in the extracellular fluid space. Low-grade BCa is characterized by ordered tumor cells with slightly larger nuclei, thus resulting in a stable nuclear-to-cytoplasmic ratio. Conversely, high-grade BCa displays marked variations in nuclear size and the nuclear-to-cytoplasmic ratio, thus suggesting significant differences in the extracellular fluid between cancers of different grades [16]. These findings suggest that lower T2 values are associated with the presence of high-grade BCa.

Radiomic features are a group of high-throughput features extracted from images that enable deeper exploration. Studies on radiomic features have developed rapidly, and several previous studies have reported the value of radiomic models for differentiating low- and high-grade BCa based on conventional computer tomography (CT) or MRI [17,18]. However, no study has used radiomic features extracted from T2 maps to predict the histological grade of BCa. Considering that T2 maps provide more accurate T2 values than conventional T2-weighted images (T2WI), and that mean time is an indicator of cell density similar to apparent diffusion coefficient (ADC) maps, we hypothesized that radiomic models based on T2 mapping could capture additional information on the morphology and texture patterns of tumors compared with those captured in conventional MR images [19,20].

Therefore, this study aimed to investigate the potential of quantitative T2 mapping for evaluating the histological grade of BCa. Additionally, we aimed to assess whether radiomic features derived from T2 mapping could enhance the diagnostic value of conventional MRI for BCa.

## 2. Materials and Methods

The study was conducted in accordance with the Declaration of Helsinki, and was approved by the Institutional Board of the West China Hospital of Sichuan University (No. 2022-IRB-339). Informed consent was obtained from all study participants between May 2021 and September 2022. Individuals suspected of having BCa were initially enrolled and underwent bladder MRI, including T2 mapping and diffusion-weighted imaging (DWI). The inclusion criteria were as follows: no prior neoadjuvant treatment (such as chemotherapy or radiation therapy), pathologically confirmed urothelial carcinoma of the bladder, and bladder lesions with a maximum diameter >1 cm.

The exclusion criterion was non-urothelial carcinoma confirmed by pathological re-sults, such as benign lesions and squamous or neuroendocrine carcinoma. All patients underwent maximal tumor resection or radical cystectomy after MR examinations. The histological grade was determined according to the 2004 World Health Organization/International Society of Urological Pathology classification [16].

### 2.1. Image Acquisition

Imaging was performed using a 3.0 T scanner (Elition, Philips Healthcare, Best, The Netherlands) with a 32-channel phased-array surface coil. All participants were instructed to urinate 2 h before the MRI examination and were not allowed to drink or urinate thereafter until examination completion. The MR protocol comprised the following sequences: T2-weighted fast spin echo sequence, DWI, axial T2 mapping, and T1-weighted three-dimensional spoiled gradient echo dynamic contrast-enhanced (DCE) sequence after Gadoteridol injection. For DWI, three *b*-values (*b* = 0, 800, and 1000 s/mm^2^) were acquired, and ADC maps were calculated using *b* = 0 and *b* = 1000. Axial T2-mapping images were acquired using the T2-prepared single-shot True FISP. The detailed scan parameters are listed in Table S1. The total MRI acquisition time was approximately 40 min.

### 2.2. Image Recognition and Feature Extraction

Three-dimensional regions of interest in bladder tumors were manually delineated separately on the T2-mapping images and DWI images using the software ITK-SNAP (version 3.8.0; www.itksnap.org, accessed on 12 June 2019) by two radiologists (LY and CLH with 5 and 2 years of experience, respectively) with blinded image information. In cases in which the participants had multifocal tumors, the tumors with the largest volume were selected for analysis. A coordinator was assigned to review the lesions marked by the radiologists and ensure consistency between the lesions and corresponding pathological results. We analyzed the agreement between the segmented outcomes obtained by different radiologists by calculating the Dice coefficients. Failure cases were defined as Dice coefficients <0.9. In those instances, the two radiologists had to re-delineate the ROI under the direction of a third radiologist (YJ with 20 years of experience) until the Dice coefficient reached 0.9. We used the image feature extraction software Python package (pyradiomics, vesrion 3.0.1) to obtain 104 radiomic features from the ROIs (in the T2 maps) and the ADC values (using the same ROIs delineated on DWIs). In each case, we extracted 208 radiomic features based on the original images, including 14 shapes, 18 histograms, and 72 texture features (Table S2). Different features had different means and variances, and Z-score normalization was performed for all the features.

### 2.3. Feature Selection and Model Construction

Following standardization, intraclass correlation coefficients (ICCs) were calculated between the features extracted by radiologists 1 and 2. Features with ICCs ≥ 0.75 were considered robust and were included in the analysis. Initially, a Pearson correlation analysis was conducted to identify and exclude radiomic features with high collinearity. Subsequently, an *F*-test was conducted for each feature and its corresponding label. The features were ranked based on their *F*-values with respect to the histological grade, and the top 50 features were selected. The least absolute shrinkage and selection operator (LASSO) algorithm was applied to determine additional prognostic features. To ensure appropriate feature selection and prevent overfitting, a 20-round, five-fold cross-validation was performed to confirm the optimal number of extracted features. Finally, logistic regression was employed to develop the prediction models.

### 2.4. Statistical Analysis

The extracted features were used to construct predictive models to determine the pathological grade of bladder tumors. The diagnostic performance of each model was evaluated using the receiver operating characteristic (ROC) curve and the area under the curve (AUC). The cut-off values for sensitivity and specificity estimation were determined based on Youden’s index.

Regarding the clinical characteristics, descriptive data are presented as frequencies and percentages, whereas parametric variables are expressed as mean ± standard deviation. Nonparametric variables are reported as means (interquartile ranges (IQRs)). Categorical variables were analyzed using Pearson’s Chi-square test or Fisher’s exact test. Continuous variables were compared using the Mann–Whitney U test or Student’s *t*-test. Findings were statistically significant when *p* < 0.05. All statistical analyses were performed using the R software (version 3.8.0; R Project for Statistical Computing, www.r-prject.org, accessed on 6 June 2023).

## 3. Results

### 3.1. Comparison of the Clinical Characteristics between the Training and Test Groups

**Figure 1:**
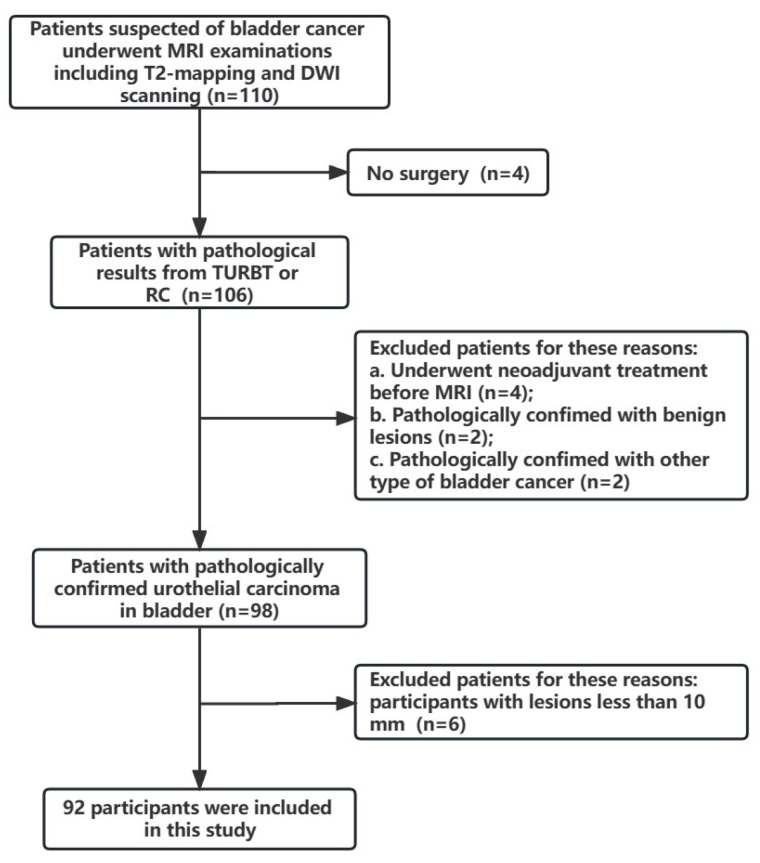
Workflow of patient selection. RC, radical cystectomy; TURBT, transurethral resection of bladder tumor; DWI, diffusion-weighted imaging.

As shown in Figure 1, 92 patients were enrolled in this study and randomly divided into the training (*n* = 64) and testing (*n* = 28) groups. There were no significant differences in the clinical characteristics between the training and testing groups in terms of age, sex, number of tumors, tumor size, and histological grade. The detailed clinical characteristics of the training and testing groups are listed in Table 1.

### 3.2. Comparisons of the T2 and ADC Values between High- and Low-Grade Cancer

**Figure 2:**
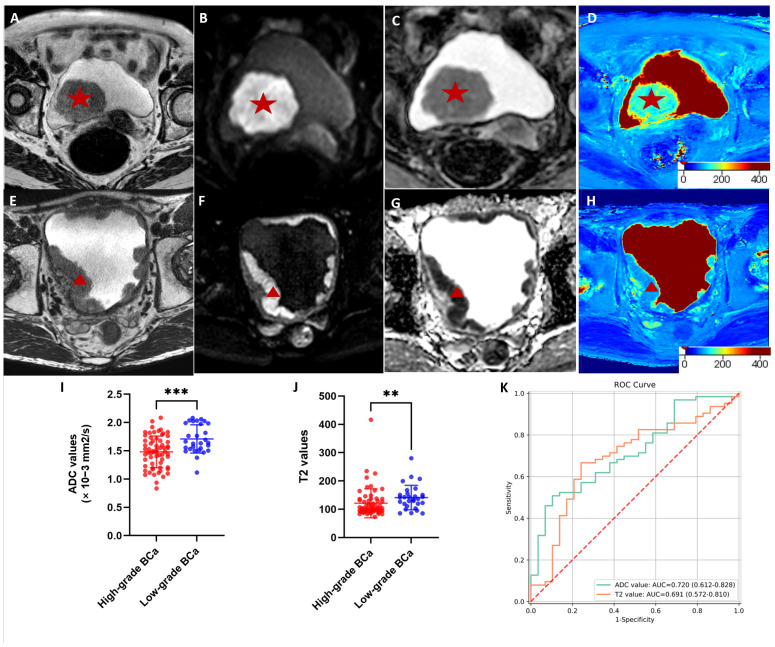
MRI scans of bladder cancer with different histologic grade, of which pathologically confirmed low-grade tumors are presented in images (**A**–**D**), and high-grade tumors are presented in images (**E**–**H**) (red stars and arrows indicate tumor location). Axial (**A**,**E**) T2-weighted image, (**B**,**F**) diffusion-weighted image (*b* = 1000 s/mm^2^), (**C**,**G**) apparent diffusion coefficient (ADC) image, (**D**,**H**) T2-mapping image fused with T2-weighted image. Scatter plots of the (**I**) ADC values and (**J**) T2 values between high- and low-grade bladder cancer. *** *p* < 0.001, ** *p* < 0.01; (**K**) receiver operating characteristic curves of models using T2 and ADC values for differentiating low- and high-grade bladder cancer. Red dotted line is the reference line.

The T2 and ADC values used to evaluate the histological grade are summarized in Figure 2. High-grade BCa had significantly lower T2 values than low-grade BCa (103.5 [IQR, 94.1–131.8] vs. 136.3 [114.8–153.0]; *p* = 0.003). The ADC values of high-grade BCa were also significantly lower than those of low-grade BCa (1.481 × 10^−3^ mm^2^/s ± 0.28 vs. 1.71 × 10^−3^ mm^2^/s ± 0.25; *p* < 0.001). We used ADC and T2 values separately to construct prediction models to discriminate the histological grade of BCa. The prediction perfor-mance of each model is shown in Figure 2K, where the ADC achieved an AUC of 0.72 (95%CI: 0.61–0.83), and the AUC of the T2 value was 0.69 (95%CI: 0.57–0.81).

### 3.3. Diagnostic Performance Outcomes of the Radiomic Models Based on T2 Mapping and ADC Images in Evaluating Histologic Grade

**Figure 3:**
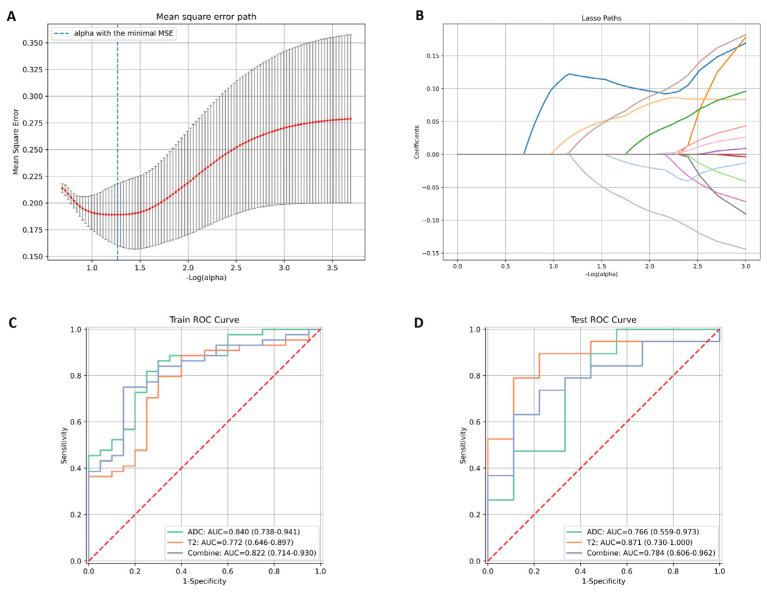
Radiomic feature selection, and prediction model construction and validation. (**A**) The selection of the tuning parameter (alpha) in the LASSO model using 20-fold cross-validation with a minimum criterion. (**B**) The coefficient of features are shown by colored lines. The dotted vertical lines on (**A**,**B**) confirm the optimal α values and the most significant features with nonzero coefficients to construct the radiomic models. Receiver operating characteristic (ROC) curves of the radiomic models based separately on T2 mapping and ADC maps, and combined radiomic features under linear discriminant analyses in the training (**C**) and testing (**D**) cohort.

A total of 104 radiomic features were extracted from the ADC and T2-mapping images of each patient. After conducting a Pearson correlation analysis and applying the LASSO algorithm to select the optimized features, 15 features from the T2-mapping images and 12 features from the ADC images with nonzero coefficients were finally chosen. Subsequently, a logistic regression algorithm was employed to construct different radiomic models, as shown in Figure 2B and Figure 3A.

The diagnostic performance of each radiomic model is presented in Figure 3C,D, and the calibration and decision curves are shown in Figure S1. First, a T2 model was developed using only radiomic features from T2 mapping. In the testing group, the AUC for distinguishing low- and high-grade cancer was 0.87 (95% confidence interval [CI]: 0.73–1.0), with a sensitivity of 0.89 and specificity of 0.78. For the ADC model constructed using only radiomic features from the ADC maps, the AUC was 0.77 (95%CI: 0.56–0.97) in the testing group, with a sensitivity of 0.79 and specificity of 0.56. Additionally, the combined model, which integrated T2 and ADC features, did not demonstrate an improvement in differentiating histological grades compared with the T2 and ADC models. It yielded an AUC value of 0.78 (95%CI: 0.61–0.96) in the testing group, with a sensitivity of 0.79 and a specificity of 0.56, and the DeLong test yielded no significant difference in AUCs among these models (*p* > 0.05).

## 4. Discussion

In this study, we preliminarily evaluated the diagnostic potential of T2 mapping and DWI for differentiating between high- and low-grade BCa. Our results revealed that the direct measurement of T2 and ADC values had poor diagnostic performance. Subsequently, we developed a radiomic model using the radiomic features from T2 maps and ADC images. The T2-mapping-based radiomic model yielded better diagnostic performance than the ADC-based and combined radiomic models.

Our results showed that T2 values exhibited moderate performance in differentiating the histological grade of BCa, whereas the radiomic model based on T2 mapping had significantly higher diagnostic performance. A previous study used the VI-RADS to predict the histologic grade of BCa and found that lesions with T2W scores of 1 or 2 were more likely to be low-grade cancers [21], thus indicating that lesions with higher T2WI signals than the muscle layer were more likely to be low-grade cancers. However, our results showed that some high-grade cancers could yield high signals, similar to low-grade cancers on T2WI, possibly because of the different histological variants of urothelial carcinoma. For example, BCa with glandular differentiation is characterized by the presence of intratumoral tubular or enteric gland-like spaces filled with high-grade columnar cells and necrotic lumen. These tissue components can yield high-signal intensities on T2WI when they constitute a major proportion of the tumor [22,23]. Conversely, our observation of a significantly improved performance using the radiomic model suggests that it incorporates tumor appearance, margins, and texture features, thus making it more representative of tumor characteristics. Considering histological variant urothelial carcinoma accounts for 10–25% of all BCa cases, and is more frequent in advanced stages with poorer chemotherapy and radiotherapy responses [22], we believe that the preoperative prediction of histological variants could also be potentially helpful in making treatment decisions. However, we failed to associate the MRI manifestations and different histological variants because we had limited cases with different histological variants, as the previous study reported [22].

Our findings revealed that the T2-mapping model outperformed both the ADC and joint models, with AUCs of 0.77 and 0.78, respectively. Our findings diverged from those of previous studies that reported superior grading performance based on DWI or ADC compared with traditional T2WI (AUCs of 0.81 vs. 0.77 and 0.78, respectively) [18,24]. This disparity can be attributed to the limitations of conventional T2WI, which relies on qualitative image interpretation using arbitrary signal intensity units. Consequently, T2WI is more suitable for lesion detection and differentiation from peritumoral tissues. In contrast, T2 mapping enables the voxel-wise evaluation of tissue composition, including interstitial edema and extracellular space expansion [14,15]. Direct quantification of T2 signals has demonstrated advantages over conventional T2WI in assessing organ functions, such as myocardial changes and renal parenchyma after transplantation [14], as well as in detecting various cancers, such as colorectal carcinoma, breast cancer, glioblastoma, and renal carcinoma [25,26]. However, to the best of our knowledge, T2 mapping has not been explored in BCa.

Although there were no significant differences between the T2 mapping and ADC radiomic models regarding the AUC value, the T2 mapping exhibited a higher negative predictive value (0.80 vs. 0.60). This result could be attributed to the influence of the T2 shine-through effect on the ADC values. Infiltrative tumors with loosely arranged cells may lead to the filling of the intercellular space by urine, thus leading to the attenuation of ADC values. The visual assessment of bladder DWI and ADC measurements using a b-value of 1000 s/mm^2^ may be limited by the high-signal intensity of urine due to the T2 shine-through effect, which could be decreased using ultrahigh b-values; however, it could degrade the image quality and is time consuming [27,28]. Conversely, quantitative T2 mapping has shown higher reproducibility and better diagnostic performance than DWI in studies on the prostate, liver, and malignant lymph nodes [19,26,29].

This study has several limitations. First, the number of participants was relatively small, and the study was conducted at a single center. Therefore, this study needs to be validated in multiple institutions with larger sample sizes. Second, 68.5% of the patients had high-grade BCa and 38% had MIBC, which was higher than the reported rates of high-grade BCa and MIBC. The most likely reason for this was that we recruited this cohort from a hospital center. Therefore, external validation of this prediction model is required. Third, our study only classified BCa into high and low grades based on the WHO 2004/2016 grading system, because pathological results from medical records only followed this single guideline. However, a recent study showed that the four-tier combination of both WHO 1973 and WHO 2004/2016 proved to be superior in predicting the prognosis of BCa [30]. Therefore, further study should be designed with pathological results following both systems. Furthermore, we did not report the histological variant, because TURBT could not adequately identify the relative proportion of variant histology in the resected specimen, which was one of the main factors affecting MRI manifestations. Finally, a basic T2-mapping sequence is time consuming, and we present the scan layers to grade seven; therefore, some large tumors were evaluated regionally. Compared with DWI, the acquisition time of T2 maps may be a major limitation in clinical practice. However, with the wide application of the deep-learning reconstruction of T2 maps [24,25], we believe that further optimization of T2 mapping could be more time efficient and provide more accurate information than conventional T2 mapping for BCa.

## 5. Conclusions

In conclusion, we developed a novel model that combines a new MR sequence with a radiomic strategy for the preoperative assessment of tumor grade in BCa. This integrated model demonstrated good diagnostic performance in distinguishing between high- and low-grade BCa. The incorporation of this model provides more comprehensive information on histological grades for BCa management.

## Figures and Tables

**Table 1:** The clinicopathological data in the training and validation groups.

Variables	Training Group (*n* = 64)	Testing Group (*n* = 28)	*p*
Age (years, mean ± SD)	66.1 ± 11.2	67.3 ± 12.0	0.264
Gender			0.505
Male	54 (15.6)	23 (82.1)	
Female	10 (84.4)	5 (17.9)	
Number of lesion			0.332
Single	39 (60.9)	15 (53.6)	
Multiple	25 (39.1)	13 (46.4)	
Tumor size (mm, mean ± SD)	26.5 ± 15.0	30.6 ± 17.6	0.663
Stage			0.475
<T2	39 (60.9)	18 (64.3)	
≥T2	25 (39.1)	10 (35.7)	
Histologic grade			0.810
High	45 (70.3)	18 (64.3)	
Low	19 (29.7)	10 (35.7)	

SD, standard deviation.

## Data Availability

The raw data supporting the conclusion of this article will be made available by the authors, without undue reservation.

## Supplementary Materials

The following supporting information can be downloaded at: https://www.mdpi.com/article/10.3390/jcm12185900/s1, Figure S1: Calibration and decision curves of radiomic models; Table S1: Tabulated overview of MR imaging parameters.; Table S2: 104 radiomic features extracted from the original MR images.
