# Supplementary material for: Radiomic Analysis of Quantitative T2 Mapping and Conventional MRI in Predicting Histologic Grade of Bladder Cancer
Source: J Clin Med. 2023 Sep 11;12(18):5900. doi: 10.3390/jcm12185900 (PMC10531568; doi:10.3390/jcm12185900)
Supplement: Supplementary file 1 [file jcm-12-05900-s001.zip › jcm-2501631-Supplementary.pdf]

Journal of
Clinical Medicine

Table S1. Tabulated overview of MR imaging parameters.

Sequence	T2-mapping	DWI
Scan plane	Axial	Axial
Voxel size (mm)	1×1.26×4	2.5×2.5×3.5
Number of slices	18	24
Slice thickness (mm)	4	3.5
TR/TE (ms)	3969/20	3738/64
NSA	1	1
FOV (mm)	200×200×79	160×160×92
Flip angle (°)	80	90
Matrix	200×159×18	252×233×24
Fat suppression	No	Yes
Parameter map type	T2 map	ADC map
Phase encoding direction	R>>L	R>>L

DWI, diffusion-weighted imaging; TR/TE, repetitive time/ echo time; NSA, number of signal averaged; FOV, field of view; ADC, apparent diffusion coefficient.

Table S2. 104 radiomic features extracted from the original MR images.

shape_Elongation_original
shape_Flatness_original
shape_LeastAxisLength_original
shape_MajorAxisLength_original
shape_Maximum2DDiameterColumn_original
shape_Maximum2DDiameterRow_original
shape_Maximum2DDiameterSlice_original
shape_Maximum3DDiameter_original
shape_MeshVolume_original
shape_MinorAxisLength_original
shape_Sphericity_original
shape_SurfaceArea_original
shape_SurfaceVolumeRatio_original
shape_VoxelVolume_original
firstorder_10Percentile_original
firstorder_90Percentile_original
firstorder_Energy_original
firstorder_Entropy_original
firstorder_InterquartileRange_original
firstorder_Kurtosis_original
firstorder_Maximum_original
firstorder_MeanAbsoluteDeviation_original

firstorder_Mean_original
firstorder_Median_original
firstorder_Minimum_original
firstorder_Range_original
firstorder_RobustMeanAbsoluteDeviation_original
firstorder_RootMeanSquared_original
firstorder_Skewness_original
firstorder_TotalEnergy_original
firstorder_Uniformity_original
firstorder_Variance_original
glcm_Autocorrelation_original
glcm_JointAverage_original
glcm_ClusterProminence_original
glcm_ClusterShade_original
glcm_ClusterTendency_original
glcm_Contrast_original
glcm_Correlation_original
glcm_DifferenceAverage_original
glcm_DifferenceEntropy_original
glcm_DifferenceVariance_original
glcm_JointEnergy_original
glcm_JointEntropy_original
glcm_Imc1_original
glcm_Imc2_original
glcm_Idm_original
glcm_Idmn_original
glcm_Id_original
glcm_Idn_original
glcm_InverseVariance_original
glcm_MaximumProbability_original
glcm_SumEntropy_original
gldm_DependenceEntropy_original
gldm_DependenceNonUniformity_original
gldm_DependenceNonUniformityNormalized_original
gldm_DependenceVariance_original
gldm_GrayLevelNonUniformity_original
gldm_GrayLevelVariance_original
gldm_HighGrayLevelEmphasis_original
gldm_LargeDependenceEmphasis_original
gldm_LargeDependenceHighGrayLevelEmphasis_original
gldm_LargeDependenceLowGrayLevelEmphasis_original

gldm_LowGrayLevelEmphasis_original
gldm_SmallDependenceEmphasis_original
gldm_SmallDependenceHighGrayLevelEmphasis_original
gldm_SmallDependenceLowGrayLevelEmphasis_original
glrlm_GrayLevelNonUniformity_original
glrlm_GrayLevelNonUniformityNormalized_original
glrlm_GrayLevelVariance_original
glrlm_HighGrayLevelRunEmphasis_original
glrlm_LongRunEmphasis_original
glrlm_LongRunHighGrayLevelEmphasis_original
glrlm_LongRunLowGrayLevelEmphasis_original
glrlm_LowGrayLevelRunEmphasis_original
glrlm_RunEntropy_original
glrlm_RunLengthNonUniformity_original
glrlm_RunLengthNonUniformityNormalized_original
glrlm_RunPercentage_original
glrlm_RunVariance_original
glrlm_ShortRunEmphasis_original
glrlm_ShortRunHighGrayLevelEmphasis_original
glrlm_ShortRunLowGrayLevelEmphasis_original
glszm_GrayLevelNonUniformity_original
glszm_GrayLevelNonUniformityNormalized_original
glszm_GrayLevelVariance_original
glszm_HighGrayLevelZoneEmphasis_original
glszm_LargeAreaEmphasis_original
glszm_LargeAreaHighGrayLevelEmphasis_original
glszm_LargeAreaLowGrayLevelEmphasis_original
glszm_LowGrayLevelZoneEmphasis_original
glszm_SizeZoneNonUniformity_original
glszm_SizeZoneNonUniformityNormalized_original
glszm_SmallAreaEmphasis_original
glszm_SmallAreaHighGrayLevelEmphasis_original
glszm_SmallAreaLowGrayLevelEmphasis_original
glszm_ZoneEntropy_original
glszm_ZonePercentage_original
glszm_ZoneVariance_original
ngtdm_Busyness_original
ngtdm_Coarseness_original
ngtdm_Complexity_original
ngtdm_Contrast_original
ngtdm_Strength_original

Figure S1

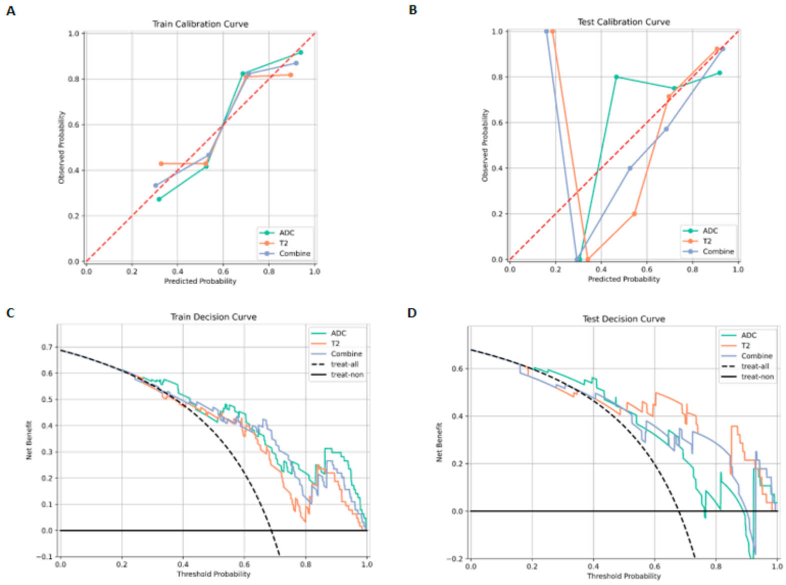

A
Train Calibration Curve
Observed Probability
Predicted Probability
ADC
T2
Combine
B
Test Calibration Curve
Observed Probability
Predicted Probability
ADC
T2
Combine
C
Train Decision Curve
Net Benefit
Threshold Probability
ADC
T2
Combine
treat-all
treat-non
D
Test Decision Curve
Net Benefit
Threshold Probability
ADC
T2
Combine
treat-all
treat-non
